# Molecular subversion of Cdc42 signalling in cancer

**DOI:** 10.1042/BST20200557

**Published:** 2021-07-01

**Authors:** Natasha P. Murphy, Ana Masara binti Ahmad Mokhtar, Helen R. Mott, Darerca Owen

**Affiliations:** 1 Department of Biochemistry, University of Cambridge, 80 Tennis Court Road, Cambridge CB2 1GA, U.K.; 2 Bioprocess Technology Division, School of Industrial Technology, Universiti Sains Malaysia, 11800 Penang, Malaysia

**Keywords:** cancer, Cdc42, metastasis, Rho GTPases

## Abstract

Cdc42 is a member of the Rho family of small GTPases and a master regulator of the actin cytoskeleton, controlling cell motility, polarity and cell cycle progression. This small G protein and its regulators have been the subject of many years of fruitful investigation and the advent of functional genomics and proteomics has opened up new avenues of exploration including how it functions at specific locations in the cell. This has coincided with the introduction of new structural techniques with the ability to study small GTPases in the context of the membrane. The role of Cdc42 in cancer is well established but the molecular details of its action are still being uncovered. Here we review alterations found to Cdc42 itself and to key components of the signal transduction pathways it controls in cancer. Given the challenges encountered with targeting small G proteins directly therapeutically, it is arguably the regulators of Cdc42 and the effector signalling pathways downstream of the small G protein which will be the most tractable targets for therapeutic intervention. These will require interrogation in order to fully understand the global signalling contribution of Cdc42, unlock the potential for mapping new signalling axes and ultimately produce inhibitors of Cdc42 driven signalling.

## Introduction

The human homologue of *Saccharomyces cerevisiae* CDC42 (cell division control protein 42) was identified in 1990 [1,2] and Cdc42 joined the Rho family of GTPases. Cdc42, together with Rac1 and RhoA, was amongst the first Rho family GTPases to be characterized both structurally and biologically [3]. The structures of these proteins in complex with their regulator and effector proteins provided the molecular details of the biological activity of these classical Rho GTPases [4–6]. Rho family proteins are found in all eukaryotes and 20 genes encoding Rho proteins have been identified in humans.

**Figure 1.**
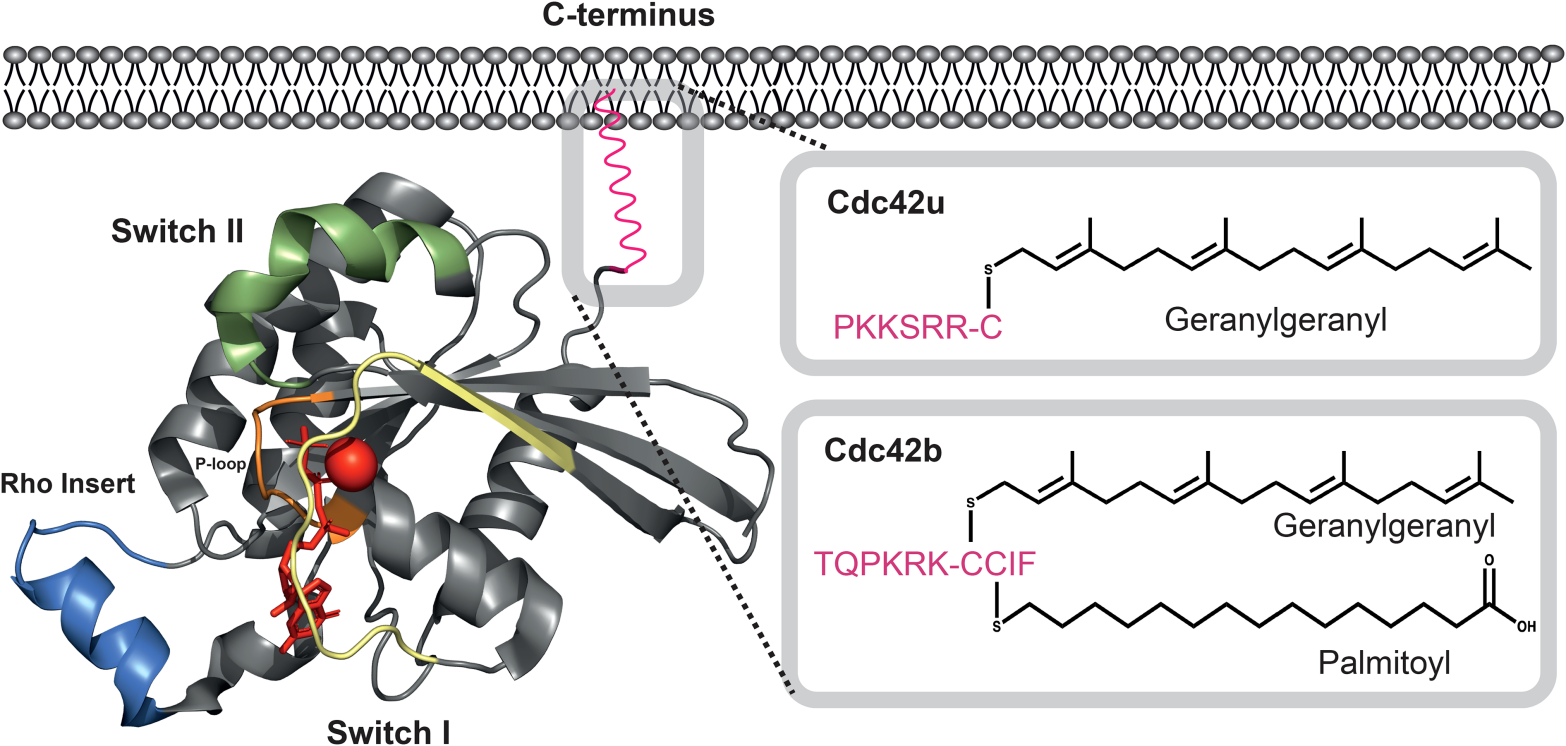
The structure of Cdc42. Cartoon representation of Cdc42 (PDB:1NF3) with key features highlighted (left): P-loop (orange, residues 10–15), Switch I, (pale yellow, residues 28–40), switch II, (green, residues 60–70), the Rho insert loop (blue, residues 122–135), the C-terminal region (magenta) encompassing a polybasic region and a CAAX box (where C represents a conserved cysteine) which is a site for post-translational lipid modification. The Mg^2+^ ion is shown as a red sphere and the bound nucleotide (GMPPNP) is shown as sticks in red. The lipid modifications for membrane anchoring of Cdc42 comprise a prenyl (geranylgeranyl) group and a palmitoyl group for the brain isoform (right).

Structurally, Cdc42 has all the key features of a Rho family GTPase (Figure 1): a P-loop (residues 10–15), two switch regions (switch I, residues 28–40 and switch II, residues 60–70), a Rho insert region (residues 122–135), a C-terminal polybasic region and a CAAX box (where C represents a conserved cysteine, Cys188 in Cdc42) which is the site for post-translational geranylgeranylation. Two splice variants of Cdc42 have been identified, which differ in their final exon. The ubiquitously expressed and more widely studied isoform, Cdc42u, was originally isolated from placenta, while a second isoform (Cdc42b) is restricted to brain. The two differ in their C-terminal ten amino acids, with Cdc42u terminating in a polybasic region ^183^KKSRR^187^ followed by CVLL, while the brain isoform, Cdc42b, has ^183^QPKRK^187^ preceding CCIF (Figure 1). The presence of two cysteines in the CAAX motif of Cdc42b results in the potential for this isoform to be lipidated by both a stable prenyl and by a reversible palmitate group, bypassing C-terminal proteolysis and carboxymethylation [7]. The two isoforms have recently been shown to have distinct functions in neurogenesis [8].

**Figure 2.**
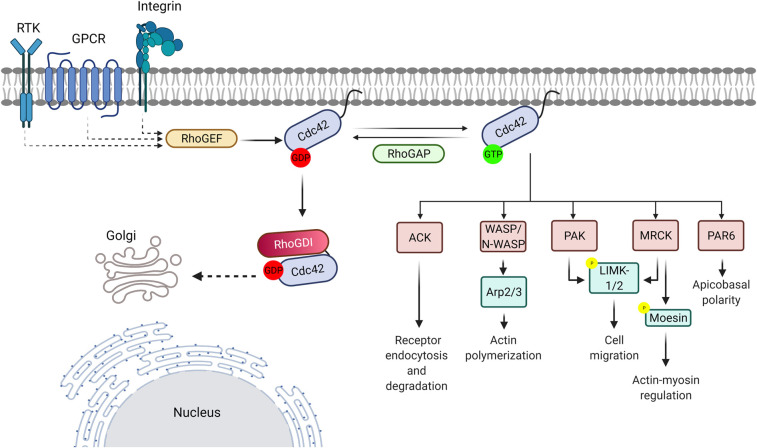
The regulation of Cdc42. Extracellular receptors (for example, TRPV4, EGFR, RET and CD44) activate Cdc42 signalling. The signals activate GEF proteins, which catalyze nucleotide exchange, resulting in the production of Cdc42·GTP. GAP proteins, which assist with hydrolysis of the GTP nucleotide, switch off the signal by promoting the formation of Cdc42·GDP. RhoGDI proteins can sequester Cdc42u in the cytoplasm, stabilizing the inactive GDP-bound form but can also act as chaperones, shuttling Cdc42u between the plasma membrane and the Golgi apparatus. Cdc42-effector interactions modulate key cellular processes including regulation of actin dynamics and polymerization, vesicle trafficking, receptor endocytosis and degradation, cell motility, actin-myosin regulation and apicobasal polarity.

The activity of Cdc42 is dictated by the bound nucleotide and regulated by three sets of regulatory proteins (Figure 2). Cdc42 activation is controlled by guanine nucleotide exchange factors (GEFs), which facilitate the exchange of GDP to GTP [9]. Cdc42 is however a hydrolase and the hydrolysis of GTP to GDP inactivates the protein, with the reaction facilitated by GTPase activating proteins (GAPs) [9]. Additionally, the activity of Cdc42u is modulated by RhoGDI proteins [10]. RhoGDIs act as inhibitors, by binding to the switch regions inhibiting nucleotide exchange and to the lipid modifications on the GTPase preventing membrane attachment. However, RhoGDI proteins also act as chaperones, ensuring that Cdc42 is delivered to the correct subcellular localization and protecting it from degradation [11,12].

Cdc42 can adopt two configurations, defined by the bound nucleotide, allowing it to function as a bimodal molecular switch and control downstream signalling pathways. Interestingly the GDP and GTP bound forms of Cdc42 adopt near identical structural states, unlike many other small G proteins including Ras [13]. In particular, in the absence of an effector protein, switch I occupies a very similar position in both forms [14]. Yet, the binding of effector proteins remains exclusive to Cdc42·GTP and results in the induction of conformational changes within the protein, resulting in an active signalling complex [14].

Membrane localization of Cdc42 is crucial for its downstream signalling and is dependent on multiple factors including lipid modification, proteolytic processing and the ability to interact with the RhoGDI chaperones [7]. Localization to specific subdomains of the relevant membranes is also critical for signalling. Nanoclustering of Cdc42 on the plasma membrane has been observed in yeast, in conjunction with an enrichment of phosphatidylserine [15]. It has also been demonstrated that phospho-4,5-bisphosphate (PIP_2_) interacts with the C-terminal di-arginine motif of Cdc42u, acting as another localization signal within membranes [16].

Cdc42 is well documented to play an important role in malignancies due to its key physiological functions. Cdc42 participates in the regulation of cytoskeletal and microtubule dynamics, transcription, cell cycle progression, cell polarity, apoptosis, phagocytosis, vesicle trafficking and cell adhesion, leading to roles in tumourigenesis as well as invasion and metastasis. Inhibition of Cdc42 and components of its signalling pathways are therefore attractive therapeutic targets in cancer and are currently the subject of small molecule and biologic based targeting efforts, which we have recently reviewed [17]. Here we review alterations found to Cdc42 itself and to key components of the signal transduction pathways it controls in cancer.

## Cdc42 alterations in cancer

**Figure 3.**
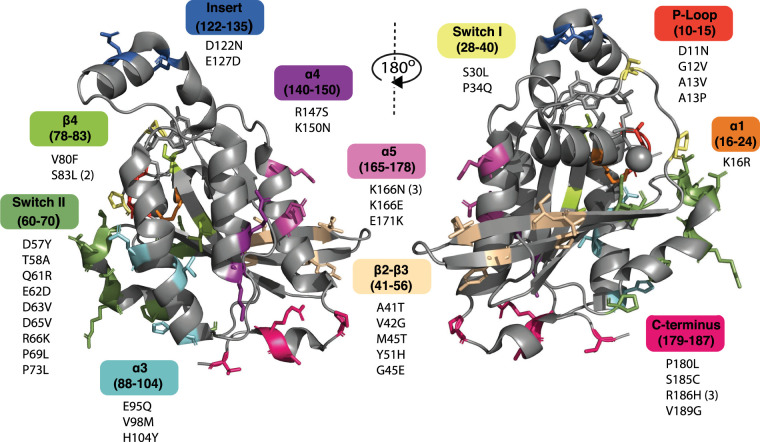
Cancer mutations in Cdc42. COSMIC mis-sense mutations shown as sticks mapped onto individual amino acid residues on the structure of Cdc42 (PDB:1NF3), colour coded by structural region. The structure of Cdc42 is shown in two orientations, rotated by 180° around the vertical axis. Numbers in brackets after mutations indicate the number of incidences documented: mutations with no associated number are found once. Data accessed January 2021 from the COSMIC v91 whole genome screening database, representing the TCGA dataset. All cancer types are included.

Relatively few Cdc42 mutations have been reported in cancer and there have been no recurring driver ­mutations identified in the gene [18]. Most mutations have been observed in only a single sample and some of these will represent passenger mutations. The mutations recorded in COSMIC representing the TCGA dataset are shown mapped onto the structure of Cdc42 in Figure 3. We have also included three mutations recorded in COSMIC outside the TCGA dataset, which have been studied widely in the literature [19,20]. Not all of these mutations are expected to increase the activity of Cdc42; indeed, some of them are predicted to decrease signalling or affect localization. This is similar to the activating and deactivating RhoA mutations that have been found in lymphomas [21].

Most of the biological consequences of the Cdc42 mutations identified in cancer have not been determined but it is useful to consider what the manifestation of these mutations could be in terms of function. Some of them (D11N, G12V [20], A13V, A13P and K16R) fall within the P-loop, a conserved, nucleotide-binding region of the protein. Gly12 is critical for GAP-assisted GTP hydrolysis and is conserved across the Ras superfamily. Mutation to any other residue except Pro at this position activates Ras proteins. Mutation at Gly12 will decrease GAP-assisted hydrolysis, locking Cdc42 in the active form and increasing output from Cdc42 regulated signalling pathways. A recent molecular dynamics simulation (MD) study highlighted solvent exposure of the nucleotide-binding site in Cdc42 G12V, suggesting that the mutation has the same effect observed for Ras proteins [22]. Mutations at residue 13 also occur in Ras and likely interfere with GAP-assisted hydrolysis due to their proximity to an essential arginine (the ‘arginine finger’) of the GAP, leading to activation. Although mutations at residue 11 have been studied less, the same argument would likely hold true. Lys16 is conserved across the Ras superfamily and contacts the β and γ phosphates of GTP. An Arg at this position could make the same contacts but the bulkier guanidino group could either increase the size of the nucleotide-binding pocket creating a fast-cycling mutant (similar to the known transforming mutant P29S in Rac1 [23]) or could prevent the γ phosphate being positioned correctly for the hydrolysis reaction.

Two of the mutations, S30L and P34Q, fall within switch I of Cdc42, which classically interacts with downstream effector proteins. Mutations in this region would be expected to alter downstream signalling from Cdc42. Pro34 is known to form key contacts with downstream effectors of Cdc42, for example, the β-hairpin of the PAK GBD [6] and a conserved YYR motif in IQGAP [24]. Mutations here may decrease effector binding but could also increase affinity in some complexes and therefore alter effector preference and Cdc42 signalling outcome. MD simulations indicate that a hydrogen bond forms from the Mg^2+^ ion via a water molecule to Pro34 of Cdc42·GDP to lock its conformation [25]. Hence Pro34 is an important residue for mediating switch I conformational mobility and its mutation may alter effector interactions or activation by GEFs. The S30L mutation may also alter specificity of the DOCK family GEFs for Cdc42 (see next section), as Ser30 has been identified as critical for the Cdc42–DOCK9 interaction, forming a hydrogen bond to Gln1812^DOCK9^ [26].

A41T, V42G, M45T, Y51H and G54E fall in the region between switch I and switch II of Cdc42 and may also therefore alter downstream effector binding. For example, V42G, is likely to have a similar effect to the V42A mutant, which has been shown to disrupt binding to effector, ACK [27]. Val42 is also known to form interactions with other effectors, for example, hydrophobic contacts with Pro241 of WASP [28]. Met45 has been identified as a critical residue interacting with Ser76^PAK1^ and Gly239^N-WASP^, respectively [27]. However, the M45T mutation had little effect on the binding of ACK, PAK and WASP, so its effects *in vivo* are difficult to assess [27]. Interestingly, some of the critical determinants for binding of the Dbl family GEFs, including residues Ala41 and Gly54, are also mutated. It is therefore possible that these mutations will affect activation of Cdc42. Where levels of active Cdc42 are lower, it is likely that the balance of active Rho family GTPases controlling migration and invasion will be altered as a result, leading to perturbation of these finely balanced systems (see later) [29].

A large number of the mutated residues found in cancers, (D57Y, T58A, Q61R [19], E62D, D63V, D65V, R66K, P69L, P73L) lie within or next to switch II, a region contributing to both effector and regulator binding. Switch II is highly charged and any changes to the charge distribution may affect effector binding, including WASP, PAK and ACK [5,27]. It is likely that the removal of negative charge in mutants D57Y, D63V and D65V will disturb key electrostatic interactions. For example, Glu62, Asp63 and Arg66 all make key contacts with the second armadillo repeat of the effector FMNL2 via salt bridges and polar interactions [30]. Glu61 is highly conserved across the Ras superfamily and a mutational hotspot in the oncogenic Ras proteins. Glu61 is the catalytic residue in GTP hydrolysis, so Q61R represents an activating mutation in Cdc42, with reduced GAP-mediated GTP hydrolysis. A number of mutants fall in the β4 and α3 regions of Cdc42: their potential functional consequences are unknown, but their positions suggest that they may affect the stability of the protein, leading to decreased signalling.

Two conservative mutations (D122N, E127D) lie in the Rho insert region, another highly charged region of the protein. Early work indicated that the Rho insert region was critical for the transforming properties of Cdc42 [31], however its molecular role remains unresolved. Whilst removal of the Rho insert region has no effect on the rate of GTP hydrolysis of Cdc42, studies have indicated that it may be an essential recognition and activation site for some effectors, such as phospholipase D [32], and can undergo significant conformational changes [32–34]. MD studies have indicated that the insert region of Cdc42 is more flexible and less helical in the GDP-bound form compared with the GTP-bound form [22]. Furthermore, in complex with RhoGDI, the insert region may undergo a reorientation of the helix and/or loss of helical structure as it has a higher temperature factor suggesting flexibility [33,34]. The insert region is also involved in binding to the scaffold protein IQGAP [24]. Asp122 and Glu127 both form salt bridges with Lys residues in IQGAP2 that are important for IQGAP dimerization. Therefore, mutations identified within the Rho insert region which alter these residues will result in ablation of key salt bridges and polar interactions with interacting partners.

R147S and K150N in α4 helix could represent mutations that alter Cdc42 membrane orientation and effector engagement. Helix α4 has been described as switch III in Ras and removal of arginine residues in Ha-Ras decreased nanoclustering of Ras at the membrane and ultimately MAPK-signalling [35]. This region may also be key to Cdc42 nanoclustering and effector engagement, although these are currently unexplored.

Mutation of Lys166^Cdc42^ is found in three cancer types: K166E, stomach; K166N, large intestine and K166N, endometrial cancer, representing a minor hotspot. Lys166 is a ubiquitination site on Cdc42, which directs proteasomal degradation [36]. This mutation would therefore be predicted to increase cellular levels of Cdc42 and consequently signalling. Lys166 also interacts with downstream effectors, for example with Pro241^WASP^ [28] and Leu77^PAK^ [6], and its mutation may also result in altered effector binding.

The final group of mutations (P180L, S185C, R186H, V189G) falls in the hypervariable region (HVR) of Cdc42u, a region regulating membrane localization [37]. Pro180 precedes the HVR and a P180L mutant may result in altered membrane localization if the HVR orientation becomes unfavourable for membrane interaction. Ser185 is a phosphorylation site on Cdc42, regulating its translocation to the cytosol by favouring its interaction with RhoGDI-1 [38], so that its mutation would affect localization of Cdc42. Arg186 is part of the di-arginine motif of Cdc42u, which mediates binding to PIP_2_-containing membranes [16]. An R186H mutation is also therefore likely to affect membrane association and potentially localization to the appropriate membrane subdomain. Interestingly, a R186C mutation resulted in aberrant palmitoylation, trapping Cdc42 in the Golgi [39]. The effect of V189G is unknown but it could affect processing or localization of canonical Cdc42 as it is juxtaposed to Cys188, the site of geranylgeranylation. Generally, HVR mutants of Ras affect nanoclustering in the membrane, altering effector recruitment and signalling, so HVR mutants in Cdc42 may have similar effects [35].

Along with mutations, gene amplifications and deletions have been documented for Cdc42 (cBioPortal). The consequences of these seem to be highly cancer type specific and still require elaboration in clinically relevant models. The top incidences of Cdc42 amplification (cBioPortal) are in endometrial carcinoma, bladder urothelial carcinoma, sarcoma and ovarian epithelial tumours, and suggest an increase in protein levels and therefore signalling. However, deletion of Cdc42 has also been linked to tumour formation in some cancer types, with one study demonstrating that deletion of Cdc42 in hepatocytes induced spontaneous hepatocellular carcinoma formation *in vivo* [40]. Intestinal deletion of Cdc42 has also been linked to hyperplasia [41].

## Oncogenic alterations to Cdc42 GEFs

While relatively few alterations to Cdc42 itself have been documented in cancer, changes to its activators, the RhoGEFs, are widely seen. Indeed many RhoGEFs were discovered as oncogenes in their own right, long before biochemical analysis revealed their regulation of Rho family nucleotide exchange [42]. Early studies led to confusion over the specificity of RhoGEFs towards their targets, with many GEFs displaying promiscuity *in vitro* [43,44]*.* Around 40 of the 80 known RhoGEFs have been linked to Cdc42 regulation and these fall into two classes: the Dbl-related proteins and the DOCK family proteins. A recent comprehensive study found 19 RhoGEFs that co-localize with Cdc42 and show activity [45], while an equally systematic investigation identified 16 RhoGEFs with activity for Cdc42 [46]. Combining these datasets identifies 28 potential Cdc42 GEFs (Tables 1 and 2).

**Table 1:** Percentage alterations for 23 Dbl Rho GEFs with Cdc42 activity across multiple cancer types from cBioPortal, TCGA datasets, Accessed January 2021

	**ESCC** ^**1**^	**OET**	**NSCLC**	**HNSC**	**UCEC**	**CESC**	**EGA**	**PAAD**	**BRCA**	**COAD**	**PRAD**	**GBM**	**M**	**LIHC**
**ARHGEF4**	0	0.51%* ^2^	1.71%^†^	1.15%^†^	4.61%^†^	1.20%^†^	1.75%^†^	0.54%*	0.73%^†^	2.18%^†^	3.23%^‡^	0.51%^†^	4.05%^†^	1.08%^†^
**ARHGEF7**	3.16%*	2.23%*	1.04%^†^	1.53%*	6.66%* ^3^	1.59%^†^	0	0.54%^†^	1.66%*	2.69%*	0.61%*	0.68%^†^	2.70%^†^	2.98%*
**ARHGEF9**	2.11%*^,†^	1.71%*	1.52%^†^	0.96%^†^	6.31%^†^	3.19%^†^	0.97%^†^	1.08%^†^	0.65%^†^	2.36%^†^	0.40%*	0.68%^†^	1.80%^†^	0.54%^‡^
**ARHGEF10**	6.32%^‡^	8.39%^‡^	6.17%^‡^	4.02%^‡^	7.00%^†^	1.59%^‡^	4.47%^†^	4.86%^‡^	4.98%^‡^	5.72%^‡^	2.17%^‡^	0.68%^†^	6.08%^†^	7.32%^‡^
**ARHGEF15**	2.11%^†^	1.03%^†^	1.71%^†^	0.96%^†^	7.34%^†^	1.99%^†^	4.47%^†^	0.54%^†^	0.65%^†^	3.20%^†^	1.21%^‡^	0.51%^†^	6.08%^†^	2.44%^‡^
**ARHGEF16**	2.11%*	2.05%*	1.04%*	0.57%^‡,^*	3.41%^†^	1.59%^†^	0	1.63%*	0.92%^‡^	1.18%^†^	0.40%*	0.68%^†^	2.70%^†^	1.63%^‡^
**ARHGEF26**	23.16%*	8.22%*	9.12%*	8.41%*	7.85%^†^	8.76%*	2.33%^†^	1.63%*	1.29%*	4.55%^†^	1.42%*	0.51%^†^	4.73%^†^	0.27%*
**ARHGEF29**	1.05%*	0.86%^‡^	0.76%^‡^	0.57%^‡,^^†^	3.24%^†^	1.20%^†,^^‡^	2.14%*	0.54%^†^	0.65%*	4.38%*	1.21%^‡^	0.17%^†,^*^,‡^	2.93%^†^	0.81%*
**FGD1**	2.11%^‡^	2.05%*	1.90%^†^	1.72%^†,^^‡^	6.48%^†^	1.20%^†^	2.53%^†^	1.09%^†^	1.20%*	2.70%^†^	0.20%*^,‡^	0.84%^†^	3.83%^†^	1.08%^‡^
**FGD2**	0	4.62%*	0.85%^‡^	0.96%^†^	3.92%^†^	0.80%^†^	1.36%*	0.54%^†^	1.11%*	2.69%^†^	0.20%^†,^^‡^	0.34%*	6.53%^†^	1.90%*
**FGD3**	1.05%*	0.68%^†^	1.71%^†^	1.34%^†^	6.48%^†^	0.80%^†^	3.50%^†^	0.54%^‡^	0.37%^†,^^‡^	1.85%^†^	0.61%*	0.51%^†,^*	2.48%^†^	1.08%^†^
**FGD4**	2.11%*	5.31%*	2.75%*	1.72%*	3.58%^†^	0.80%*	2.14%^†^	3.26%*	0.83%*	1.68%^†^	0.81%*	0.84%^†,^*	2.25%^†^	1.08%^†^
**PLEKHG1**	0	1.88%^‡^	1.80%^†^	2.87%*	7.51%^†^	2.00%^†^	4.09%^†^	1.09%^‡,^^†^	0.92%^†^	3.37%^†^	0.81%^†^	1.18%^†^	5.63%^†^	0
**PLEKHG2**	0	8.56%*	3.80%*	1.52%^†^	7.34%^†^	4.38%*	2.14%*	8.15%*	2.12%*	3.20%^†^	0.40%*	0	6.53%^†^	1.08%*
**PLEKHG3**	1.05%^†^	0.34%^†^	2.47%^†^	1.34%^†^	5.46%^†^	1.20%^†^	3.31%^†^	0.54%^†^	0.46%*	2.02%^†^	0.61%*	0.84%^†^	6.08%^†^	1.08%^†^
**PLEKHG4**	2.11%*	2.05%^‡^	2.09%^†^	1.15%^†^	6.14%^†^	3.19%^†^	2.33%^†^	0	1.29%^‡^	2.02%^†^	0.40%^†^	0.68%^†^	6.31%^†^	0.54%^†^
**PREX2**	3.16%*	5.99%*	6.74%^†^	3.25%^†^	7.51%^†^	1.99%^†^	12.2%^†^	3.80%*	5.17%*	6.73%^†^	6.48%*	0.84%^†^	22.5%^†^	6.50%*
**MCF2 (DBL)**	4.21%*	2.23%*	3.99%^†^	2.29%^†^	10.2%^,†^	2.39%^†^	2.72%^†^	1.63%*	0.74%*	4.38%^†^	0.81%^‡^	0.34%*^,†^	9.68%^†^	0.81%^†^
**MCF2L (DBS)**	4.21%*	2.57%*	2.09%^†^	1.91%*	5.29%^†^	2.79%^†^	3.11%^†^	1.63%^†^	1.75%*	3.70%^†^	0.81%^†,^^‡^	0.68%^†^	5.18%^†^	2.71%*
**RASGRF2**	2.11%^‡^	3.08%^‡^	3.51%^†^	1.91%^†^	7.17%^†^	1.99%^†^	3.31%^†^	1.09%^†^	0.74%^†^	4.04%^†^	1.82%^‡^	1.01%^†^	6.98%^†^	1.36%^†^
**VAV2**	2.11%*	0.85%^†^	1.80%^†^	0.96%*^,†^	5.63%^†^	1.20%^†^	2.14%^†^	0.54%^‡^	0.55%^†^	1.85%^†^	1.21%*	0.68%*	3.60%^†^	1.36%*
**FARP1**	12.10%^†^	1.88%*	1.52%^†^	1.91%^†^	5.97%^†^	1.99%^†^	3.89%^†^	0.54%*^,†^	1.75%*	2.86%^†^	0.61%^‡^	0.34%^†^	5.63%^†^	2.98%*
**TUBA**	2.11%^†^	1.20%^†^	2.09%^†^	2.29%^†^	7.85%^†^	2.39%^†^	3.31%^†^	1.09%^†^	1.01%^†^	3.54%*	1.21%^‡^	0.68%^†^	8.78%^†^	0.81%*

1 Cancer types key: Cervical Squamous Cell Carcinoma (CESC), Colorectal Adenocarcinoma (COAD), Uterine Corpus Endometrial carcinoma (UCEC), Esophageal Squamous Cell Carcinoma (ESCC), Esophagogastric Adenocarcinoma (EGA), Glioblastoma (GBM), Liver Hepatocellular Carcinoma (LIHC), Head and Neck squamous cell carcinoma (HNSC), Invasive Breast Carcinoma (BRCA), Melanoma (M), Ovarian Epithelial Tumour (OET), Pancreatic Adenocarcinoma (PAAD), Prostate Adenocarcinoma (PRAD)

2 Alteration type key:* amplification,^†^ mutation,^‡^ deep deletion.

3 Grey shading denotes a percentage alteration type above 5% for an individual RhoGEF in a single cancer type

**Table 2:** Percentage alterations for five DOCK Rho GEFs with Cdc42 activity across multiple cancer types from cBioPortal, TCGA datasets, Accessed January 2021

	**ESCC^1^**	**OET**	**NSCLC**	**HNSC**	**UCEC**	**CESC**	**EGA**	**PAAD**	**BRCA**	**COAD**	**PRAD**	**GBM**	**M**	**LIHC**
**DOCK7**	1.05%*^2^	2.23%*	3.04%^†^	2.68%^†^	10.5%^†^ 3	2.39%^†^	3.89%^†^	1.09%^†^	1.48%^†^	4.21%^†^	1.01%^‡^	1.01%^†^	8.56%^†^	1.63%^†^
**DOCK8**	4.21%^‡^	2.40%*	2.66%^†^	2.68%*	10.4%^†^	2.39%^†^	3.70%^†^	1.09%^†^	1.38%*	5.05%^†^	1.01%^‡^	1.35%^†^	12.84%^†^	2.44%^†^
**DOCK9**	3.16%^†^	2.23%*	2.75%^†^	1.53%^†^	9.56%^†^	2.79%^†^	3.50%^†^	1.63%^†^	1.75%*	5.05%^†^	0.81%^‡^	0.51%^†^	10.59%^†^	3.52%*
**DOCK10**	3.16%^†^	1.37%^†^	7.12%^†^	2.10%^†^	11.9%^†^	3.98%^†^	6.42%^†^	2.17%*	1.75%^†^	4.48%^†^	1.01%^†^	1.18%^†^	10.81%^†^	2.71%^†^
**DOCK11**	2.11%*	1.37%^†^	4.37%^†^	3.25%^†^	13.6%^†^	5.58%^†^	4.09%^†^	1.09%^†^	2.31%^†^	4.55%^†^	0.81%^†^	1.01%^†^	11.04%^†^	1.08%^†^

^1^Cancer types key: Cervical Squamous Cell Carcinoma (CESC), Colorectal Adenocarcinoma (COAD), Uterine Corpus Endometrial carcinoma (UCEC), Esophageal Squamous Cell Carcinoma (ESCC), Esophagogastric Adenocarcinoma (EGA), Glioblastoma (GBM), Liver Hepatocellular Carcinoma (LIHC), Head and Neck squamous cell carcinoma (HNSC), Invasive Breast Carcinoma (BRCA), Melanoma (M), Ovarian Epithelial Tumour (OET), Pancreatic Adenocarcinoma (PAAD), Prostate Adenocarcinoma (PRAD)

^2^Alteration type key:* amplification,^†^ mutation,^‡^ deep deletion.

^3^Grey shading denotes a percentage alteration type above 5% for an individual RhoGEF in a single cancer type

**Table 3:** Percentage alterations for 25 RhoGAPs with Cdc42 activity across multiple cancer types from cBioPortal, TCGA datasets, Accessed March 2021

	**ESCC**	**OET**	**NSCLC**	**HNSC**	**EC**	**CESC**	**EGA**	**PAAD**	**BRCA**	**COAD**	**PRAD**	**GBM**	**M**	**LIHC**
**ARHGAP1**	1.05%*	1.03%*	0.66%^†^	1.53%*	1.37%*	0.80%*	1.94%^†^	1.09%*	0.65%*	0.67%^†^	1.01%^‡^	0.34%^‡^	1.58%^†^	0
**ARHGAP5**	3.16%^†^	1.03%*	3.04%*	1.72%^†^	9.22%^†^	3.59%^†^	4.86%^†^	1.63%^†^	1.38%*	4.71%^†^	0.81%^†^	0.34%^†^	6.76%^†^	0.81%^†^
**ARHGAP11b**	1.05%*	1.88%^‡^	1.71%^‡^	0.38%*	2.22%^†^	0.40%^‡^	0.78%^†^	0.54%^†,^^‡^	1.85%^‡^	1.35%^‡^	0.81%*^,‡^	0.17%^‡,^*	1.35%^‡^	0.54%^‡^
**ARHGAP17**	0	1.03%*	2.47%^†^	2.10%^†^	7.00%^†^	1.20%^†^	2.53%^†^	1.09%^†^	3.41%*	1.68%^†^	1.21%*	0.34%^†^	4.50%^†^	0.54%^†^
**ARHGAP20**	1.05%^†^	1.20%*	2.28%^†^	1.72%^†^	8.02%^†^	3.19%^‡^	3.31%^†^	0.54%^†^	1.11%^‡^	3.7%^†^	0.61%^‡^	0.51%*	5.63%^†^	0.81%^†^
**ARHGAP21**	3.16%^†^	2.91%*	3.13%^†^	1.91%^†^	10.9%^†^	1.99%^†^	2.72%^†^	1.63%^†^	1.20%^†^	5.05%^†^	0.81%^†^	1.1%^†^	8.11%^†^	1.08%*
**ARHGAP22**	1.05%*^,‡^	0.68%*	0.95%^†^	0.76%*	6.48%^†^	0.80%^‡†^	1.95%^†^	0	0.37%*	4.04%^†^	0.81%^‡^	0.51%^‡^	6.98%^†^	1.63%^†^
**ARHGAP30**	2.11%*	2.05%*	5.41%*	1.72%^†^	6.66%^†^	2.39%*	4.28%^†^	3.26%*	9.23%*	2.86%^†^	0.61%^‡,^*	1.01%^†^	8.78%^†^	10.0%*
**ARHGAP31**	5.26%*	2.40%*	3.51%^†^	2.68%*	8.19%^†^	3.19%^†^	4.47%^†^	1.63%^†^	1.57%^†^	4.88%^†^	0.40%^†,^*	0.68%^†^	8.11%^†^	1.63%^†^
**ARHGAP32**	3.16%*	2.23%*	4.18%^†^	1.34%^†^	10.1%^†^	3.19%^‡,^^†^	3.70%^†^	1.63%^†^	0.74%^‡^	3.54%^†^	0.81%^†^	1.52%^†^	9.68%^†^	1.36%^†^
**SRGAP2**	1.05%*	2.74%*	2.94%*	0.57%^†^	5.63%^†^	1.99%^†^	1.75%*	1.63%*	8.30%*	2.86%^†^	0.61%*	0.84%*	3.6%^†^	6.23%*
**ARHGAP39**	12.63%*	25.86%*	4.65%*	7.84%*	3.92%*	3.19%*	5.45%*	8.15%*	10.3%*	3.20%*	6.28%*	1.01%*	6.53%^†^	9.21%*
**ARHGAP40**	0	1.71%*	0.95%*	0.57%*	1.19%*	1.20%*	2.92%*	0	1.11%*	7.24%*	0.40%*^,‡^	0.17%*	0.90%*	0.27%*
**GMIP**	2.11%*	4.62%*	1.61%^†^	1.72%^†^	5.29%^†^	1.20%^†^	3.11%^†^	0.54%	0.74%*	1.35%^†^	0.61%^†^	0.51%^†^	3.83%^†^	1.36%*^,†^
**FAM13B**	1.05%^†^	0.86%*	0.47%^†^	0.76%^†^	5.97%^†^	0.80%^†^	1.56%^†^	1.63%^†^	0.74%^†^	1.18%^†^	0.61%*^,‡^	0.34%^†^	4.05%^†^	0.81%^†^
**MYO9B**	2.11%^†^	7.88%*	2.56%^†^	2.49%^†^	7.85%^†^	2.39%^†^	2.92%^†^	2.17%^†^	1.57%*	3.70%^†^	1.21%^†^	1.18%^†^	6.76%^†^	2.71%^†^
**STARD8**	2.11%*	1.20%*	2.75%^†^	1.15%*	7.51%^†^	1.99%^†^	2.53%^†^	0.54%^†,‡,^*	0.75%^†^	2.86%^†^	0.61%*	0.51%^†^	5.41%^†^	0.54%^†^
**PIK3R1**	2.11%^‡^	3.08%^‡^	1.42%^†^	1.53%^†^	28.0%^†^	3.98%^†^	3.11%^†^	0.54%^†^	2.58%^†^	5.39%^†^	3.85%^‡^	6.42%^†^	3.60%^†^	1.08%^†^
**PIK3R2**	1.05%*	4.96%*	1.42%^†^	0.96%^†^	4.10%^†^	0.40%*^,†,‡^	2.14%^†^	0.54%^†^	1.20%*	1.68%^†^	0.61%^†^	0.68%^†^	2.48%^†^	1.90%*
**DEPDC1B**	3.16%^‡^	3.08%^‡^	1.61%^†^	1.14%^‡^	4.44%^†^	0.80%^†^	2.14%^‡^	0	1.20%^‡^	1.18%^†^	3.24%^‡^	0.34%^†^	2.70%^†^	0.54%^†,^*
**SYDE1**	1.05%^†,^*	11.1%*	0.66%^†^*	0.38%*^,†^	3.92%^†^	1.59%^†^	1.95%^†^	0.54%^†^	2.40%*	2.19%^†^	0.61%*	0.51%*	1.13%^†^	1.08%*^,†^
**OPHN1**	3.16%^‡^	1.20%*	2.94%^†^	0.96%^†,^^‡,^*	7.85%^†^	1.59%^†^	1.75%^†^	0.54%*^,‡^	0.55%*	1.18%^†^	0.81%*	1.01%^†^	4.05%^†^	0
**ARAP1**	20.00%*	5.14%*	1.99%*	4.59%*	6.66%^†^	1.99%^†^	2.92%^†^	1.63%^†^	3.87%*	4.71%^†^	0.81%^†^	0.34%*	5.18%^†^	0.81%^†^*
**ARAP2**	3.16%^†^	0.68%^†^	4.27%^†^	1.34%^†^	8.87%^†^	2.39%^†^	4.47%^†^	0.54%^†^	0.65%^†^	5.89%^†^	0.40%^‡^	0.84%^†^	14.2%^†^	0.54%^†,^*
**ARAP3**	0	1.03%*	2.94%^†^	1.53%^†^	9.22%^†^	2.39%^†^	3.11%^†^	1.09%*	1.11%^†^	4.71%^†^	0.61%*^,†,^^‡^	0.68%^†^	5.63%^†^	1.89%^†^

1 Cancer types key: Cervical Squamous Cell Carcinoma (CESC), Colorectal Adenocarcinoma (COAD), Uterine Corpus Endometrial carcinoma (UCEC), Esophageal Squamous Cell Carcinoma (ESCC), Esophagogastric Adenocarcinoma (EGA), Glioblastoma (GBM), Liver Hepatocellular Carcinoma (LIHC), Head and Neck squamous cell carcinoma (HNSC), Invasive Breast Carcinoma (BRCA), Melanoma (M), Ovarian Epithelial Tumour (OET), Pancreatic Adenocarcinoma (PAAD), Prostate Adenocarcinoma (PRAD).

2 Alteration type key:* amplification,^†^ mutation,^‡^ deep deletion.

3 Grey shading denotes a percentage alteration type above 5% for an individual RhoGAP in a single cancer type

**Table 4:** Percentage alteration for three RhoGDIs of multiple cancers from cBioPortal. Accessed: March 2021

	**ESCC**1	**OET**	**NSCLC**	**HNSC**	**UCEC**	**CESC**	**EGA**	**PAAD**	**BRCA**	**COAD**	**PRAD**	**GBM**	**M**	**HC**
**RhoGDI-1**	-	3.42%*^2^	1.52%*	0.38%^‡^^,†,^*	2.73%*	0.79%^‡^	0.78%^†^	0.54%^†,^*	3.60%*	0.67%*	0.40%*	0.34%^†^	2.48%*	4.34%*
**RhoGDI-2**	3.16%*	3.77%*	0.66%^‡^	0.38%*	2.22%^†^	0.40%^†^	0.97%^‡^	2.72%*	0.92%*	1.01%^†^	1.21%^‡^	0.68%*	1.35%^†^	0.27%^‡^
**RhoGDI-3**	-	1.71%*	0.38%^‡^	0.19%*	0.85%^‡,^*	0.40%^‡^^,†^	1.75%^‡^	0.54%^‡^	3.69%*	0.51%^‡^	1.01%*	0.34%*	1.13%^†^	0.81%^‡^

1 Cancer types key: Cervical Squamous Cell Carcinoma (CESC), Colorectal Adenocarcinoma (COAD), Uterine Corpus Endometrial carcinoma (UCEC), Esophageal Squamous Cell Carcinoma (ESCC), Esophagogastric Adenocarcinoma (EGA), Glioblastoma (GBM), Liver Hepatocellular Carcinoma (LIHC), Head and Neck squamous cell carcinoma (HNSC), Invasive Breast Carcinoma (BRCA), Melanoma (M), Ovarian Epithelial Tumour (OET), Pancreatic Adenocarcinoma (PAAD), Prostate Adenocarcinoma (PRAD)

2 Alteration type key:* amplification,^†^ mutation,^‡^ deep deletion.

**Table 5:** Percentage alterations for Cdc42 downstream effector proteins, representing every effector family (WASPs, PAKs, TNKs, Cdc4EP1s/BORGs, MRCKs, MLKs, SPECs, IQGAPs, PARs, Formins) across multiple cancer types from cBioPortal, TCGA datasets, Accessed March 2021

	**ESCC^1^**	**OET**	**NSCLC**	**HNSC**	**UCEC**	**CESC**	**EGA**	**PAAD**	**BRCA**	**COAD**	**PRAD**	**GBM**	**M**	**LIHC**	**UDSA**
**WASP**	4.21%^‡ 2,3^	5.31%*	1.42%^†^	1.91%^‡^	5.11%^†^	1.20%*	0.97%^‡^	0.54%*^,†^	1.11%^†^	1.18%^†^	0.61%*	0.34%^†^	2.93%^†^	1.08%^‡^	7.69%^‡^
**PAK2**	27.37%*	15.58%*	15.0%*	12.81%*	6.31%*	14.74%*	4.67%*	2.72%*	2.40%*	1.35%^†^	2.43%^‡^	1.01%*	2.52%^†^	0.81%*	15.38%^†^
**ACK**	28.42%*	15.92%*	14.25%*	12.81%*	6.83%*	15.14%*	5.06%*	1.63%*	2.31%*	2.52%^†^	2.02%^‡^	0.84%*	6.08%^†^	1.08%*	0
**CEP4**	0	1.54%*	1.99%*	0.96%^†^	1.54%*	1.20%*^,†^	1.95%*	1.63%*	4.52%*	1.35%^†^	0.61%*	0.34%^†^	2.93%^†^	3.52%*	0
**MRCKα**	2.11%^†,^*	4.97%*	3.61%^†^	1.34%^†^	7.68%^†^	3.19%^†^	3.50%^†^	1.63%*	8.95%*	4.21%^†^	1.01%^‡^	0.34%*	6.53%^†^	5.15%*	0
**MLK4**	2.11%*	4.97%*	2.75%*	0.76%^†^	4.78%^†^	1.20%^†^	3.11%^†^	2.72%^†^	9.04%*	5.05%^†^	1.42%^‡^	1.01%^†^	9.91%^†^	6.23%*	0
**SPEC1**	2.11%*	7.19%*	7.41%*	0.96%*	6.83%*	2.39%*	3.70%*	2.72%*	9.04%*	0.67%*	1.62%*	0.51%*	2.03%*	10.84%*	0
**IQGAP3**	2.11%^†^	3.77%*	5.03%*	2.29%^†^	7.85%^†^	1.99%*	3.31%^†^	3.80%*	7.93%*	3.70%^†^	0.61%*	0.84%^†^	8.33%^†^	10.57%*	0
**Par6b**	1.05%*^,†^	3.94%*	1.71%*	0.19%*^,†^	2.90%*	1.20%*	4.47%*	1.09%*	2.86%*	6.57%*	1.21%*	0.34%^†^	1.35%^†^	1.08%*	7.69%*
**mDia2**	2.11%^‡^	1.37%^‡^	3.32%^†^	2.29%^†^	7.51%^†^	2.00%^‡^	3.31%^†^	0.54%^†^	0.74%^‡,^^†^	3.20%^†^	7.09%^‡^	0.84%^†^	5.63%^†^	1.63%^‡^	15.38%^†^

1 Cancer types key: Cervical Squamous Cell Carcinoma (CESC), Colorectal Adenocarcinoma (COAD), Uterine Corpus Endometrial carcinoma (UCEC), Esophageal Squamous Cell Carcinoma (ESCC), Esophagogastric Adenocarcinoma (EGA), Glioblastoma (GBM), Liver Hepatocellular Carcinoma (LIHC), Head and Neck squamous cell carcinoma (HNSC), Invasive Breast Carcinoma (BRCA), Melanoma (M), Ovarian Epithelial Tumour (OET), Pancreatic Adenocarcinoma (PAAD), Prostate Adenocarcinoma (PRAD), Undifferentiated Stomach Adenocarcinoma (UDSA)

2 Alteration type key:* amplification,^†^ mutation,^‡^ deep deletion.

3 Grey shading denotes a percentage alteration type above 5% for an individual Rho effector in a single cancer type

Amongst the Cdc42 GEFs, many Dbl family members are altered in cancers, for example Ect2, Tiam-1, Trio, P-Rex1–2 and Vav1–3 (reviewed in [47]). The DOCK family consists of 11 members and has been linked to roles in cancer migration and invasion [48]. DOCK proteins modulate switching between the two main modes of cell motility (see later) via the Rho GTPases. DOCK10, a Cdc42 GEF, is necessary for amoeboid motility [49] while DOCK3 drives mesenchymal motility and invasion in melanoma cells through Rac1 [50]. A recent analysis of TCGA data identified significantly higher expression of the RhoGEFs Trio, Net1, Ect2, Tiam2, Farp1, ARHGEF12 and BCR in primary cancer types compared with normal tissues [51]. Additionally, in gastric cancer, eight Cdc42 regulating RhoGEFs were correlated with poor patient survival: Trio, PlekHG3, ITSN1, Ect2, Tiam2, Farp1, ARHGEF10, ARHGEF12 [51].

For the 28 potential Cdc42 GEFs [45,46], amplification and mutation are the most frequently reported types of alteration across all cancer types (Tables 1 and 2). For the Cdc42-active Dbl proteins, oesophageal squamous cell carcinoma, endometrial carcinoma and melanoma show the highest mutational alterations (Table 1). The DOCK Family GEFs are also mutated, particularly in melanoma and in endometrial carcinoma (Table 2), which often displays a high mutational burden due to increased DNA mismatch repair [52].

## Cdc42 GAP alterations in cancer

Another key family of Cdc42 regulatory proteins are the GAP proteins. As GAPs catalyze the hydrolysis of GTP to GDP and act as negative regulators they should be tumour suppressors. However, RhoGAPs have been associated with both pro- and anti-proliferative functions and their role is nuanced and complex within specific Rho GTPase signalling pathways. The RhoGAPs are thought to be relatively promiscuous [46], although a number had been suggested to be Cdc42 exclusive, including ARHGAP1, ARHGAP17 and ARHGAP31 [45]. In a recent survey, however, none of the 50 GAPs tested had activity restricted to Cdc42, although 16 were identified as regulating Cdc42 alongside RhoA and/or Rac1 [45]. A complementary analysis documented a further 11 Cdc42 GAPs [46], giving a total of 25 RhoGAPs active against Cdc42.

Where there are alterations to these 25 RhoGAPs, amplification and mutation are most frequently reported (Table 3). Mutations in the highly conserved arginine finger of the GAP domain are rare but some have been reported, including: ARHGAP11b (R87W (2)), ARHGAP5 (R1297L/C/S), ARHGAP17 (R288Q), ARHGAP30 (R55H (2)), ARHGAP31 (R56W), STARD8 (R608H), SYDE1 (R436H) and OPHN1 (R409S). Mutation of this residue would prevent GAP-assisted GTP hydrolysis and result in sustained activation of Cdc42.

Studies into the biological relevance of some RhoGAP domain missense mutants have focused on the DLC (deleted in liver cancer) family of GAPs, established tumour suppressors found down-regulated or deleted in cancer [53,54]. DLC3 (STARD8) has been identified as a Cdc42 regulator whose levels are reduced, at the mRNA level at least, in kidney, lung, ovarian, uterine and breast cancer [53]. Missense mutations are relatively common in the *DLC* genes. 7/9 mutations in the GAP domain of DLC1, identified in cBioPortal, showed decreased GAP activity *in vitro*, while mutations in two other regions also resulted in less biological activity due to reduced interactions with cellular partners [55]. Hence, mutations that reduce the GAP activity of RhoGAPs are associated with cancer, but other mutations affecting intramolecular interactions, scaffolding functions or subcellular localization, also contribute to the oncogenic process.

Amplification of the Cdc42 GAPs however would increase GAP activity and could serve to selectively deactivate Cdc42. This would affect the fine balance of active Rho family proteins and potentially perturb the functions that rely on this, for example, cell motility. Alternatively, the non-GAP functions of these proteins may also make an important contribution to oncogenic pathways.

In terms of the global contribution of RhoGAPS to cancer, several studies have recently identified a role for RhoGAPs in regulating cell morphology. Depletion of 15 RhoGAPs in MCF10A resulted in a significant change in morphological parameters associated with epithelial-mesenchymal transition (EMT), including a change to elongated spindle-like morphologies [56]. Amongst these, ARHGAP43 (SH3Bp1) and ARHGAP4 were identified as major regulators of cell morphology. Depletion of SH3Bp1 has previously been linked to a loss of spatial control of Cdc42 activity in epithelial junction formation and enhanced growth of filopodia [57], linking this Cdc42 GAP to increased cancer cell migration.

## Cdc42–RhoGDI alterations in cancer

Three proteins comprise the RhoGDI family, which act as multi-functional regulators of Rho family GTPases. RhoGDIs can extract Rho GTPases from membranes, burying the G protein prenyl moiety into the hydrophobic pocket of their C-terminal Ig domain. The RhoGDI proteins therefore control the subcellular localization of Cdc42 and a subset of other Rho GTPases [58–61]. They also act as chaperones, regulating the levels of Rho family GTPases and maintaining a balance between the individual members of the family relative to one another [62,63]. Finally, RhoGDIs are conventional negative regulators, preventing nucleotide exchange on their targets by binding to the switch regions of Rho family GTPases via their N-terminal domain [64].

The specificity of the RhoGDI proteins is key to their regulation of Rho GTPase signalling pathways within cancer but the molecular basis behind their selectivity is not well understood. Cdc42 has been found to interact with both RhoGDI-1 and -2 [58,65], although it is preferentially regulated by RhoGDI-1 [66]. Loss of RhoGDI-1 stimulates constitutive activation of Cdc42, causing increased COX-2 activity, promoting breast cancer development [67]. RhoGDI-3 is the least well studied of the RhoGDI family in terms of its Cdc42 interaction. It differs from the other RhoGDI proteins by an N-terminal extension, thought to be involved in Golgi localization [61]. Depletion of RhoGDI-3 has been linked to increased amoeboid movement in diffuse large B-cell lymphoma [68]. In terms of alterations in cancer, all three RhoGDIs show aberrant expression in a range of cancers, although at relatively low levels (Table 4). cBioPortal data indicates that alteration frequencies across all cancer types are highest in RhoGDI-1, followed by RhoGDI-3 and finally RhoGDI-2. All three proteins show amplifications, mutations and deep deletions.

The RhoGDIs are regulated by post-translation modifications including phosphorylation, ubiquitination and sumoylation, and by phospholipids and protein–protein interactions [9]. For example, RhoGDI-1 phosphorylation at Tyr156 by Src promotes Cdc42 activation [69], which is reversed by dephosphorylation of the same residue by PTP-PEST [70]. Any mutations that ablate the Cdc42–RhoGDI-1 interaction will likely result in aberrant Cdc42 activation [58]. For instance, R66A or R68A mutations within the Cdc42 switch II region selectively interfere with its interaction with RhoGDI, resulting in the hyperactivation of Cdc42 [34]. Mutations occurring within the hydrophobic, isoprene binding, cleft of RhoGDIs, such as I177M, will also disrupt the binding between RhoGDIs and Cdc42 [71]. Besides the I177M mutation, several other mutations are recorded in cBioPortal in the Ig domain such as L196R, R180H and K113Q, identified in glioblastoma, breast and colorectal cancers, respectively.

## Cdc42 effector alterations in cancer

The involvement of Cdc42 in both tumourigenesis and metastasis is reliant on its effector proteins. There are currently 32 known Cdc42 effectors and Table 5 summarizes the alterations found in the Cdc42 effectors most commonly associated with various cancer types.

**Figure 4.**
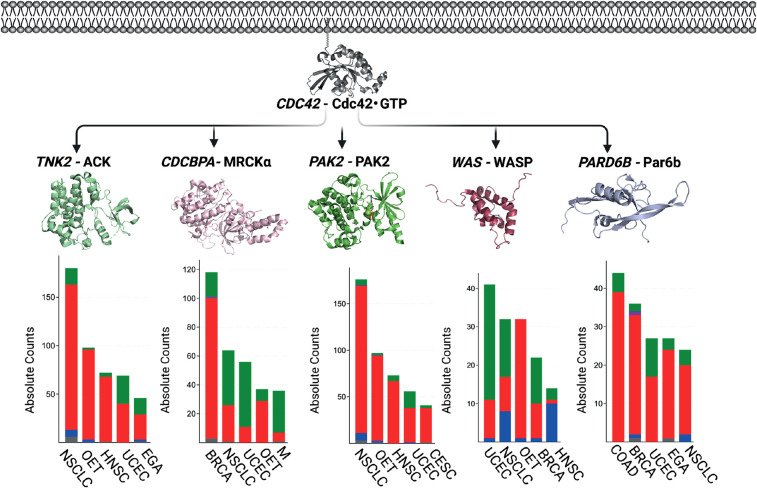
Cdc42 effector alterations in cancer. Alterations of a selection of Cdc42 effector genes *TNK2* (ACK), *CDCBPA* (MRCKα), *PAK2* (PAK2), *WAS* (WASP) and *PARD6B* (Par6b) for the five cancer types with the highest incidence, where the Y axes represent absolute counts of incidence recorded for each gene and the X axes indicate specific cancer type. Both mutation and copy number alteration (CNA) data are shown. Green represents mutations; red, amplification; blue, deep deletion; purple, fusion and grey, multiple alteration. Data is from cBioPortal, January 2021. Key for cancer types: CESC, Cervical Squamous Cell Carcinoma; COAD, Colorectal Adenocarcinoma; UCEC, Uterine Corpus Endometrial carcinoma; EGA, Esophagogastric Adenocarcinoma; HNSC, Head and Neck squamous cell carcinoma; BRCA, Invasive Breast Carcinoma; M, Melanoma; OET, Ovarian Epithelial Tumour. Note that the bar charts utilize different vertical axis scales for clarity.

Amongst the Cdc42 kinase effectors showing the highest frequencies of alterations are the myotonic dystrophy kinase-related Cdc42-binding kinases (MRCKα and MRCKβ). Binding of Cdc42 to MRCK likely directs membrane recruitment rather than activation of kinase activity [72]. Amplifications predominate in MRCKα alterations, for example in invasive breast carcinoma (Figure 4), while MRCKβ is predominantly mutated, with the highest frequency in endometrial carcinoma (cBioPortal). Given that the C-terminal regulatory domains of MRCK proteins autoinhibit the N-terminal kinase domain, activating mutations were predicted to lie in the C-terminal regions of MRCK [73]. Four mutations have been reported C-terminal to the kinase domain of MRCKβ, including the truncating mutation R1092* found in intestinal adenocarcinoma [72]. One minor mutational hotspot occurs at Pro675 (cBioPortal) in MRCKα. P675S/T mutations are found in eight samples across invasive breast carcinoma, endometrial carcinoma, oesophageal squamous cell carcinoma and oesophagogastric adenocarcinoma. Pro675 lies within a negative autoregulatory region, which is known to interact with the kinase domain [74].

Another family of serine-threonine Cdc42 effector kinases with high alteration frequencies are the p21-activated kinases (PAKs). PAKs are mainly amplified, for example PAK2 in non-small cell lung cancer, ovarian epithelial tumour and endometrial carcinoma (Figure 4). Increased PAK1 and PAK4 activity is known to result from gene amplification [75]. Mutations do not feature largely in PAK alterations. Instead, their hyperactivation plays a major role in their contribution to cancer [75]. Hyperactivation of the PAK can result from excessive activation by Cdc42/Rac1, or by upstream miRNAs, for example miR-424 was recently found to hyperactivate the cancer stem cell pool in breast cancer through activation of PAK1 [76].

Activated Cdc42-associated kinase (ACK) is a non-receptor tyrosine kinase with additional scaffolding functions and an effector of Cdc42 [77]. ACK is predominantly subject to genomic amplifications, for example in NSCLC, ovarian epithelial tumour, HNSC and endometrial carcinoma (Figure 4). ACK mRNA overexpression is frequently observed, for example in lung, prostate, breast, pancreatic, hepatocellular and gastric carcinoma [78]. In terms of mutations, several cancer driver mutations have been identified in ACK [79], including R34L, R99Q, E346K and M409I, which result in increased kinase activity [80]. A frameshift mutation, P761Rfs*72, in endometrial carcinoma is a minor recurrent mutation, resulting in truncation of the UBA domain of ACK, which regulates the proteasomal degradation of the protein [81].

The best characterized Cdc42-controlled actin polymerization pathways regulating migration involve the Wiskott Aldrich Syndrome scaffold proteins (WASP and N-WASP). Cdc42·GTP alleviates intramolecular inhibition of WASP proteins, allowing engagement of the Arp2/3 complex [82], which nucleates actin filament growth. Activating mutations (L270P and I294T) in WASP, which relieve autoinhibition, are associated with immunodeficiency disease X-linked neutropenia (XLN), whilst loss of function mutations are associated with Wiskott–Aldrich syndrome (WAS) [83]. Both diseases have been linked to a high incidence of cancer; WAS with non-Hodgkin, Hodgkin and Burkitt lymphomas and XLN with acute myeloid leukaemia and myelodysplastic syndrome [84]. Alterations in other cancer types occur at a very low frequency (Table 4). Deletions of WASP are recorded in NSCLC and HNSC, and WASP has been shown to play a tumour suppressor role in some cancers [85].

Th role of N-WASP in metastasis has been the subject of multiple recent studies, particularly in pancreatic ductal adenocarcinoma (PDAC) [86,87]. Activation of the spindle and kinetochore-associated protein 1 (SKA1) by Cdc42 in PDAC has been correlated with poor survival and with increased N-WASP-Arp2/3 activity and actin remodelling [88]. Combining 3D matrices with microfluidics demonstrated a role for N-WASP in regulating invasive protrusion in NIH3T3 fibroblast and human osteosarcoma cell models [89]. Very few alterations are reported for N-WASP (*WASL*) in cancers. A minor mutational hotspot occurs at Val422 in gastrointestinal cancer, where frameshift results in loss of actin polymerization [90]. Another minor mutational hotspot is Arg131 in the WH1 domain, and the R131Q mutation likely affects protein–protein interactions [90]. The tumour promoting and suppressing roles for the WASP proteins in different cancer types are currently not well understood. Greater understanding of the expanding roles for the proteins in both the cytoplasm and the nucleus may reconcile these contradictions in the future [90].

The classic role of Cdc42 in regulating apicobasal polarity and sustained directionality in motility is mediated by the Cdc42–aPKC–Par6–Par3 complex, in which Cdc42 interacts with Par6 [91]. *PARD6A* mainly shows amplifications; very few mutations are reported. Overexpression of the *PARD6A* and *PARD6B* genes have been associated with increased cell proliferation, tumour initiation and epithelial-to-mesenchymal transition (EMT) in breast cancer [92,93]. Overall, the Par proteins, like the WASPs, have lower alteration frequencies compared with the Cdc42 kinase effectors (Figure 4).

Interestingly, 29 of the 32 Cdc42 effectors have endometrial carcinoma in the top five cancer types where they are found altered, suggesting understanding and potentially targeting Cdc42 signalling in this cancer could have therapeutic benefit. Relatively little is known regarding the role of Cdc42 in endometrial carcinoma, however CD73-generated adenosine underpins epithelial integrity in endometrial cancer, potentially through an A_1_R-Cdc42-N-WASP-Arp2/3 pathway [94] and prostaglandin F_2α_ involvement in endometrial cancer cell migration and adhesion is also dependent on Cdc42 [95]. Additionally, the receptor TRPV4 has been linked to metastasis in endometrial cancer via Rac1 and RhoA/ROCK signalling [96] and there is an established link for TRPV4-Cdc42-N-WASP signalling in glioblastoma migration [97].

## Cdc42 and its effectors in cancer metastasis

There is a growing body of evidence implicating Cdc42 in the formation and regulation of specialized cellular protrusions associated with invasion and metastasis, including invadopodia [98] and extracellular vesicles (EVs) [99]. EVs are now recognized to be important for transferring cargo between different tumour subpopulations and between cancer and normal cells [100]. The proteomes of invadopodia and EVs revealed a set of common proteins regulating their assembly including Cdc42, EGFR, RhoA, RhoC, Src, several subunits of the ARP2/3 complex, IQGAP1 and moesin [99]. Additionally, the growing understanding of the role of the stroma in metastasis implicates Cdc42, with the Cdc42 GAP, ARHGAP31, being highly expressed in cancer-associated stroma [101]. Increasingly, studies of pre-cancerous neoplasms aim to predict alterations which drive metastasis [102]. A role for Cdc42 in regulating angiogenesis has been proposed recently, with Cdc42 driving vascular tip migration via N-WASP in endothelial cells [103]. Abnormalities in tumour vascularization lead to hypoxia, which promotes metastasis [103].

**Figure 5.**
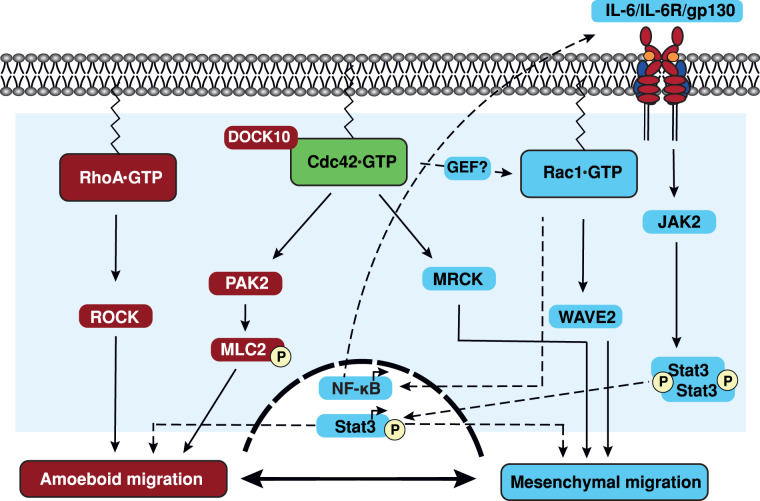
Cdc42 signalling in cell migration. Cdc42 is capable of regulating both mesenchymal migration and amoeboid migration due to the selective activation of Cdc42 by different GEF proteins. In melanoma cells, DOCK10 activates Cdc42, which can then interact with its downstream effector PAK2 to promote amoeboid migration through MLC2. The other well characterized signalling pathway which promotes amoeboid migration is RhoA activation of ROCK kinase. Mesenchymal migration is regulated by Rac1, via activation of WAVE2. Additionally, Cdc42 is proposed to regulate Rac1 activation via a currently uncharacterized GEF. Cdc42 mediated activation of MRCK proteins is also known to drive mesenchymal migration. Rac1 signalling to NFκB induces transcription of the IL-6 gene. IL-6 binds to IL-6R to activate JAK2, which phosphorylates Stat3, allowing it to dimerize and translocate to the nucleus, where it is transcriptionally active.

The complexity of Cdc42's role in metastasis can be partly attributed to the different modes of cell migration that exist: an elongated mesenchymal mode, a rounded amoeboid mode [104] and collective migration [105]. Generally, the elongated mode of migration is associated with actin polymerization and Rac1 activation, whilst the amoeboid mode is characterized by higher levels of actomyosin contractility and RhoA/ROCK signalling [104] (Figure 5). The balance between Rac1 and RhoA signalling is key to determining which mode of migration occurs under normal physiological conditions. It is known that tumour cells can switch between either mode of migration [104,106,107].

Cdc42 signalling pathways have been linked to the faster amoeboid mode of migration [49]. DOCK10 activates Cdc42 which in turn leads to phosphorylation of MLC2 by PAK2, driving the amoeboid phenotype in melanoma [49] (Figure 5). Cdc42 also controls the transcription factor Stat3, linked to increased secretion of MMP-9 into the ECM via ROCK-JAK1-Stat3 signalling, increasing invasion by amoeboid cells [108]. Additionally, negative regulators of Cdc42, the RasGRFs, have recently been suggested to be a major determinant of the transition between the two modes of migration [109]. RasGRF1/2 bind to Cdc42 via their DH domain, preventing Cdc42 activation by outcompeting Cdc42 GEFs (Dbl, Ost and DOCK10) and therefore inhibiting amoeboid motility [109]. RasGRF2 in particular, has reduced expression across multiple human cancers [110]. GM130-RasGRF also importantly controls levels of active Cdc42 at the Golgi, therefore contributing to cell polarity and directional migration in tumourigenesis [111]. Overall, down-regulation of negative regulators such as the RasGRFs, overexpression of exchange factors including DOCK10 and signalling from transcription factors downstream of Cdc42 contribute to increased amoeboid migration and a metastatic phenotype [109].

Cdc42 has also been shown to drive the second mode of migration, mesenchymal migration (Figure 5). Mesenchymal morphology and invasion have been shown to be regulated by Cdc42-MRCK signalling [112]. However, deletion of DOCK10, PAK2 or N-WASP, which all contribute to amoeboid migration in melanoma cells, also resulted in an elongated morphology, implicating more Cdc42 pathways in mesenchymal migration [49]. Although less well studied, Cdc42 is also active in collective migration [105].

## Conclusions and future perspectives

Overall, it is clear that the pathways Cdc42 controls are potential targets for therapeutic intervention in cancer, despite the lack of driver mutations identified in Cdc42 itself. There are multiple alterations found in Cdc42's regulators and effectors, particularly its activators, the GEFs, and its downstream kinases. However, despite growing indications of the importance of Cdc42 signalling axes therapeutically, the role of all of the Rho family GTPases still requires more rigorous experimental validation in clinically relevant models. There has however been progress in the application of 3D models to study the biological roles of the GTPases and their binding proteins [113,114], with Cdc42 identified as a key regulator in the formation of single cell 3D invasion tunnels [115].

The biological challenges involve targeting this key switch of multiple signalling pathways, as highlighted by the recent systems-based studies of Rho family networks [45,46]. Inhibiting a signalling node protein with functions essential to normal physiology is no small task. There is acknowledgement within the field of the need to identify which subtypes of cancer would benefit from a potent and selective Cdc42 inhibitor if one were available [17]. Nevertheless, the role of Cdc42, in migration and in regulation of specialized invadopodia and extracellular vesicles, positions it at the centre of multiple investigations into cell invasion and metastasis in different cancer types. There is clearly still more to understand including its response and contribution to the mechanisms of stromal and ECM regulation. As more receptor signalling axes are found to converge on Cdc42 controlled signalling pathways, tractable extracellular receptors are also emerging for potential therapeutic inhibition. Cdc42 and its regulatory and effector protein partners continue to demonstrate an ever-central role in the molecular subversion of signalling in cancer.

## Perspectives

- ***Importance of the field:*** Cdc42 is one of the ‘classical’ Rho GTPases and acts as a central signalling node for pathways regulating cell migration, apicobasal polarity and vesicle trafficking. A complete understanding of its structure, membrane localization, regulation and downstream signalling is essential to an assessment of its overall contribution to pathologies in different tissue types.
- ***Current thinking:*** In cancer, multiple oncogenic alterations are found in Cdc42 regulating GEFs and its downstream kinases, and Cdc42 is known to regulate signalling pathways controlling cellular processes of migration and invasion via its control of specialized invadopodia and extracellular vesicles.
- ***Future directions:*** There is more to understand of the contribution of Cdc42 to the mechanisms of cancer cell migration, invasion, stromal and ECM regulation. This will contribute to understanding the relevance of inhibiting Cdc42 signalling axes therapeutically.

## Open Access Statement

Open access for this article was enabled by the participation of University of Cambridge in an all-inclusive *Read & Publish* pilot with Portland Press and the Biochemical Society under a transformative agreement with JISC.

## Abbreviations

ACK: Activated Cdc42-associated kinase
Cdc42: cell division control protein 42
COSMIC: catalogue of somatic mutations in cancer
Dbl: diffuse B-cell lymphoma
DOCK: Dedicator of cytokinesis
ECM: extracellular matrix
EMT: epithelial, mesenchymal transition
GAP: GTPase activating protein
GDP: guanine nucleotide diphosphate
GEF: guanine nucleotide exchange factor
GTP: guanine nucleotide triphosphate
HVR: hypervariable region
MD: molecular dynamics
MRCK: myotonic dystrophy kinase-related Cdc42-binding kinases
PAK: p21 activated kinase
PDAC: pancreatic adenocarcinoma
PIP_2_: phospho-4,5-bisphosphate
Rac1: Ras-related C3 botulinum toxin substrate 1
RhoA: Ras homology family member A
RhoGAP: Rho-family GTPase activating protein domain
RhoGDI: guanine dissociation inhibitor for Rho family G proteins; guanine nucleotide-exchange factor
TCGA: the cancer genome atlas
WAS: Wiskott Aldrich Syndrome
WASP: Wiskott Aldrich Syndrome proteins
XLN: X-linked neutropenia
